# Intrathecal Infusion of Hydrogen-Rich Normal Saline Attenuates Neuropathic Pain via Inhibition of Activation of Spinal Astrocytes and Microglia in Rats

**DOI:** 10.1371/journal.pone.0097436

**Published:** 2014-05-23

**Authors:** Yanhu Ge, Feixiang Wu, Xuejun Sun, Zhenghua Xiang, Liqun Yang, Shengdong Huang, Zhijie Lu, Yuming Sun, Wei-Feng Yu

**Affiliations:** 1 Department of Anesthesiology, Eastern Hepatobiliary Surgery Hospital, Changhai Hospital, Second Military Medical University, Shanghai, China; 2 Department of Anesthesiology, 309th Hospital of CPLA, Beijing, China; 3 Department of Diving Medicine, Faculty of Naval Medicine, Second Military Medical University, Shanghai, China; 4 Department of Neurobiology, Second Military Medical University, Shanghai, China; 5 Department of Cardiothoracic Surgery, Changhai Hospital, Second Military Medical University, Shanghai, China; Imperial College London, Chelsea & Westminster Hospital, United Kingdom

## Abstract

**Background:**

Reactive oxygen and nitrogen species are key molecules that mediate neuropathic pain. Although hydrogen is an established antioxidant, its effect on chronic pain has not been characterized. This study was to investigate the efficacy and mechanisms of hydrogen-rich normal saline induced analgesia.

**Methodology/Principal findings:**

In a rat model of neuropathic pain induced by L5 spinal nerve ligation (L5 SNL), intrathecal injection of hydrogen-rich normal saline relieved L5 SNL-induced mechanical allodynia and thermal hyperalgesia. Importantly, repeated administration of hydrogen-rich normal saline did not lead to tolerance. Preemptive treatment with hydrogen-rich normal saline prevented development of neuropathic pain behavior. Immunofluorochrome analysis revealed that hydrogen-rich normal saline treatment significantly attenuated L5 SNL-induced increase of 8-hydroxyguanosine immunoreactive cells in the ipsilateral spinal dorsal horn. Western blot analysis of SDS/PAGE-fractionated tyrosine-nitrated proteins showed that L5 SNL led to increased expression of tyrosine-nitrated Mn-containing superoxide dismutase (MnSOD) in the spinal cord, and hydrogen-rich normal saline administration reversed the tyrosine-nitrated MnSOD overexpression. We also showed that the analgesic effect of hydrogen-rich normal saline was associated with decreased activation of astrocytes and microglia, attenuated expression of interleukin-1β (IL-1β) and tumor necrosis factor-α (TNF-α) in the spinal cord.

**Conclusion/Significance:**

Intrathecal injection of hydrogen-rich normal saline produced analgesic effect in neuropathic rat. Hydrogen-rich normal saline-induced analgesia in neuropathic rats is mediated by reducing the activation of spinal astrocytes and microglia, which is induced by overproduction of hydroxyl and peroxynitrite.

## Introduction

Reactive oxygen species (ROS) and reactive nitrogen species (RNS) are key factors in the pathogenesis of central nervous system (CNS) disorders. Studies have shown that ROS and the superoxide byproduct peroxynitrite (ONOO^−^) are involved in neuropathic pain [1], [2]. Free radical scavengers such as TEMPOL, phenyl-N-tert-butylnitrone (PBN) and vitamin E, could alleviate neuropathic pain [3]-[6], delivery these agents directly to the sub-arachnoid space produced more potent effects, suggesting that antioxidants exert their anti-allodynic action mainly at the spinal levels [7]. The CNS derives its energy almost exclusively from oxidative metabolism of the mitochondrial respiratory chain, and consumes a disproportionately high amount of the body’s O_2_ supply. Leakage of high energy electrons along the mitochondria electron transport chain causes the formation of superoxide (O_2_^−^), hydrogen peroxide (H_2_O_2_) and peroxynitrite [8]-[11]. Therefore, in the spinal dorsal horn where signals from primary somatosensory neurons are modulated depending on prior experience, ROS and RNS play an important role in the balance between inhibitory and facilitatory influence.

There is considerable evidence that ROS and RNS contribute to neuropathic pain. However, there remains much uncertainty as to the relative contribution of different types of ROS or RNS molecules. Free radical scavengers indeed alleviate neuropathic pain, but we have not elucidated an antioxidant with no adverse side effects for therapeutic use. Hydrogen has been identified as a ROS scavenger. Ohsawa and colleagues found that hydrogen acts as an anti-oxidant by selectively reducing hydroxyl (·OH) and peroxynitrite radicals [12]. However, the analgesic efficacy of hydrogen on neuropathic pain is unknown.

Astrocytes are the most abundant cells in CNS and were historically regarded as support cells [13]. Astrocytes contact with neural synapses closely. It is possible that astrocytes not only support the neurons but also modulate the synaptic transmission by regulating the external environment of synapse. Studies have demonstrated that astrocytes contribute to both induction and maintenance of neuropathic pain [14]–[20]. Microglia are derived from peripheral myeloid precursor cells and penetrate the CNS during embryogenesis [21]. In CNS, microglia are heterogeneously distributed, the spinal cord is one of the areas where microglial density is particularly high [22], [23]. The number of microglia dramatically increases after peripheral never injury in pain models. Numerous studies have demonstrated that microglia contribute to neuropathic pain [24]–[27]. Moreover, a series of studies have implied that the production of ROS in both astrocytes and microglia contribute to neuronal inflammatory process, neuronal death and pain disorders in the CNS [28]–[32]. Therefore, we speculated that production of ROS and RNS within spinal astrocytes and microglia may be involved in glial activation, leading to central sensitization.

In the present study, we characterized the effect of hydrogen-rich normal saline (HRS) on mechanical and thermal hyperalgesia in a rat model of neuropathic pain, in an attempt to provide evidences to confirm hydroxyl and peroxynitrite as significant mediators of glial activation and proinflammatory cytokine release in neuropathic rats. We hypothesize that elimination of hydroxyl and peroxynitrite within spinal glial cells by HRS will attenuate activation of astrocytes and microglia, prevent proinflammatory cytokine-mediated central sensitization, and thus be a potential therapy for neuropathic pain.

## Materials and Method

### Sample size, randomization and blinding

The sample size of this study was calculated using G*power 3.1 software (Universität Kiel, Germany). Randomization was performed using a computerized random number generator and animals were assigned to each group by residue method. All the behavior testing and the section analysis were performed by an investigator blinded to the origin of the animal.

### Experimental animals

Young adult male Sprague-Dawley rats (3 months old, 300–320 g, Sino-British SIPPR/BK Laboratory Animal Ltd., Shanghai, China) were housed two per cage with free access to food and water. Rats were acclimated for at least 5 days before experimental procedures. Basic foot withdrawal thresholds to mechanical stimuli and paw withdraw latencies (PWLs) to heat stimuli were tested for all rats, animals with abnormal mechanical thresholds or PWLs were excluded. All experimental procedures were approved by the Ethics Committee for Animal Experimentation of the Second Military Medical University. The protocols used in this research project complied with the guidelines for the care and use of laboratory animals of the Second Military Medical University.

### Rat model of neuropathic pain (L5 spinal nerve ligation (L5 SNL))

Rats were anesthetized with isoflurane (2% induction, 1.5% maintenance) in O_2_, and placed in the prone position. After removing the left L6 vertebral transverse process, the L5 spinal nerve on the left side was separated from the adjacent L4 spinal nerve and the L5 spinal nerve was tightly ligated using 6–0 silk thread [33].

### Intrathecal catheterization

Rats were anesthetized with isoflurane and mounted in a conventional stereotaxic frame. A midline incision was made on the skull and the superficial neck muscles were separated by a midline incision. The underlying layer of muscles were separated along the midline by blunt dissection. Next, the muscles were separated from their point of origin on the occipital bone to expose the atlanto-occipital membrane. A small incision was then made on the membrane. A 12.5 cm PE-10 tube (Becton Dickinson and Co., Franklin Lakes, NJ USA) was inserted into the spinal subarachnoid space [34]. The tip of the tube lay in the region of the lumbar enlargement. Before implantation, the tube was sterilized by immersion in 75% ethanol, followed by normal saline (NS) wash, and was filled with NS. The tube was then secured by a loop of suture material around it and through the neck muscle. The external end was sealed with a cap made of a short segment of PE-50 tube (Becton Dickinson and Co., Franklin Lakes, NJ USA) with one end closed. The foot withdrawal thresholds to mechanical stimuli and the PWLs to heat were examined after the surgery, rats developed motor/sensory problems were excluded.

### Behavioral testing

The foot withdrawal thresholds to mechanical stimuli were used as an indicator of mechanical sensitivity of the affected paw. The mechanical thresholds were measured using the “up-down” method [35]. Briefly, rats were placed in a plastic chamber (8.0×8.5×20 cm) on the top of a mesh screen platform and habituated for at least 10 min. A set of von Frey monofilaments (von Frey numbers: 3.65, 3.87, 4.10, 4.31, 4.52, 4.74, 4.92 and 5.16; equivalent to 0.45, 0.74, 1.26, 2.04, 3.31, 5.50, 8.32, and 14.45 g, respectively) was chosen. Testing was initiated with filament 4.31 (2.04 g), in the middle of the series, the filaments were applied perpendicular to the ventral surface of the base at the proximal part of the third or fourth toe (this area is the most sensitive site) with sufficient force to bend the filament slightly for 2–3 s. An abrupt withdrawal of the hind paw during stimulation or immediately after stimulus removal was considered as a positive response. If there was a positive response, the next lower filament was used, and if not, the next higher filament was applied. The test was continued until responses to six stimuli had been obtained after the first change in response. The 50% threshold value was calculated using Dixon's formula [36]: 50% threshold  = 10^(X+κδ)^/10^4^, where X is the value of the final von Frey hair used (in logarithmic units), κ is the tabular value for the pattern of positive/negative responses, and δ is the mean difference between stimuli in logarithmic units (0.22). In cases where continuous positive or negative responses were observed all the way to the end of the stimulus spectrum, values of 3.54 (0.34 g) or 5.28 (18.72 g) were assigned, respectively.

Thermal hyperalgesia was evaluated using Hargreaves' procedure [37]. Rats were placed in a plastic cage with a glass floor on the day of the experiment. After a 30 min habituation period, the plantar surface (the base at the proximal part of the third or fourth toe) of the hind paw was exposed to a beam of radiant heat delivered with an infrared bulb (Osram halogen-bellaphot bulb; 8V, 50W) through the glass floor. When paw movement interrupted the reflected light, a photoelectric cell turned off the lamp. PWLs were automatically displayed to the nearest 0.1 s, and the cut-off time was 20 s in order to prevent tissue damage. Light intensity was preset to obtain a baseline latency of approximately 11 seconds. The time in seconds between the activation of the heat source and paw withdrawal was recorded. The mean PWL was calculated from six consecutive trials (each performed every 30 min).

### Treatment with HRS

Hydrogen rich normal saline was obtained from the Department of Diving Medicine, the Faculty of Naval Medicine, Second Military Medical University. Normal saline was hydrogenated under 0.4 Mpa (1Mpa = 1×10^6^ pascal) for 6 hrs to a supersaturated level, sterilized with gamma radiation and stored under atmospheric pressure (approximately 101325 pascal in Shanghai, China) at 4°C in a sealed aluminum bag with no dead space, and used within a week [38]–[40]. Hydrogen content was 0.6 mmol/L as measured with gas chromatography [12]. For treatment, 20 ul of HRS was injected intrathecally. Control rats received an equal volume of NS.

### Immunostaining

Rats were sacrificed 1 hr after HRS or NS treatment, on the 12th day after L5 SNL or sham operation, when behavioral testing was ended. Catheterized rats received no injection were sacrificed on the 15th day after catheterization. Once rats were anesthetized, the chest was opened and the aorta was perfused with saline, followed by cold fixative (4% paraformaldehyde in 0.1 M phosphate buffer (PB, PH 7.4)). The L4–5 spinal cord segment was removed, post-fixed overnight, and then placed in 30% sucrose until equilibration. The spinal cord was cryosectioned at 16 µm, and the sections were collected in 0.1 M PB. After blocking with 2% normal human serum containing 0.3% Triton X-100 in PB for 1 hr at room temperature, all sections were incubated with a mixture of primary mouse monoclonal antibody against 8-OH-G (1∶200 Abcam, Cambridge, MA USA) and goat polyclonal antibody against GFAP (1∶200 Santa Cruz Biotechnology, Santa Cruz, CA USA) or goat polyclonal antibody against Iba 1 (1∶200 Abcam, Cambridge, MA USA) for 36–48 hrs at 4°C, followed by a mixture of donkey anti-mouse IgG Fluorescein conjugated and donkey anti-goat IgG cyanine conjugated secondary antibodies (1∶300, Jackson ImmunoResearch Laboratories, West Grove, PA, USA) for 2 hrs at room temperature. Sections were then washed, air-dried, and coverslipped with mounting medium. To confirm the specificity of the immunolabeling, some sections were processed as above, but without the primary antibodies. The sections from all animals were processed together in each staining process to minimize staining variability.

Sections were analyzed with a fluorescence microscope (Nikon corporation, Shinjuku, Tokyo Japan), and images were captured with a CCD camera. Consistency was ensured by taking all photographs at the same exposure. Digital images were analyzed using a computer image analysis system (Furi Company, Shanghai China). Ten serial sections (16 µm thick, 150 µm apart) were selected for analysis from each rat. In every image, a rectangular area was manually outlined in the dorsal horn using the rectangular marquee tool, and the size of every selected area was 50 cm×22 cm (2500 µm×1100 µm in the spinal sections). At 20-fold magnification, counts were obtained of the numbers of cells in the areas outlined in the rectangular that were positive for 8-OH-G, GFAP and Iba-1, and double staining cells in the areas were also counted. The average number of positively labeled cells was calculated and presented as data.

### Protein extraction

Animals were anesthetized and exanguinated. The lumbar spinal cord was quickly dissected, and immediately frozen. Tissues were stored at −80°C until analysis. Tissue samples (1 g) were homogenized in 5.0 ml extraction buffer (4°C) consisting of phosphate buffered saline (PBS) containing 1mM phenylmethylsulfonyl fluoride. Solubilized extracts were sonicated using a sonifier cell disruptor and centrifuged to remove cellular debris. The protein concentration was assessed using the Bicinchoninic Acid (BCA) Protein Assay method.

### Nitrotyrosine immunoprecipitation

Solubilized proteins (2 mg) were incubated with 10 ug mouse monoclonal anti-nitrotyrosine antibody (agarose conjugated, Millipore Corporation, Billerica, MA, USA). Immune complexes were precipitated overnight at 4°C, washed in extraction buffer, resuspended in sample loading buffer (16 mM Tris HCl, PH 6.8/2.5% glycerol/0.5% SDS/200 mM 2-mercaptoethanol/0.001% bromophenol blue), heated and immediately fractionated by reducing SDS/PAGE in 12% gels.

### Western blot analysis

After separation by SDS/PAGE, proteins were transferred electrophoretically (100 V, 1 hr) to nitrocellulose membranes, which were blocked (1 hr, 20°C) with 5% nonfat milk in 50 mM Tris HCl, PH 7.4/150 mM NaCl/0.05% Tween 20 (TBS/T). For detection of Mn-SOD, blots were incubated (overnight, 20°C) with mouse monoclonal anti-MnSOD antibody (1∶1000, Millipore Corporation, Billerica, MA, USA). After three times wash in TBS/T, the immunocomplexed membranes were probed with goat anti-mouse horseradish peroxidase-conjugated secondary antibody (1∶10000). Probed membranes were washed three times, and immunoreactive proteins were detected using enhanced chemiluminescence. The integrated optical density (IOD) of lanes resulting from immunoprecipitation was calculated using Image-pro plus analysis (Media Cybernetics Inc., Rockville, MD, USA).

### ELISA

Tissue samples were homogenized in a homogenization buffer and centrifugated at 40,000×g for 1 hr. The supernatant was collected, and the. IL-1β and TNF-α levels were assessed by enzyme-linked immunosorbent assay (R&D Systems, Minneapolis, MN, USA) according to the instructions of the manufacturer, and are expressed as picograms per microgram of protein.

### Statistics

Data are presented as mean±standard errors of the mean (SEM) and analyzed using the SPSS statistical program. For behavioral responses analysis, two-way repeated ANOVAs were used, followed by post hoc LSD test. Data from image-analysis, immunoprecipitation and ELISA were analyzed by one-way ANOVA analysis. P value of <0.05 was considered to be significant.

## Results

### The effect of single-dose HRS on mechanical allodynia

**Figure 1:**
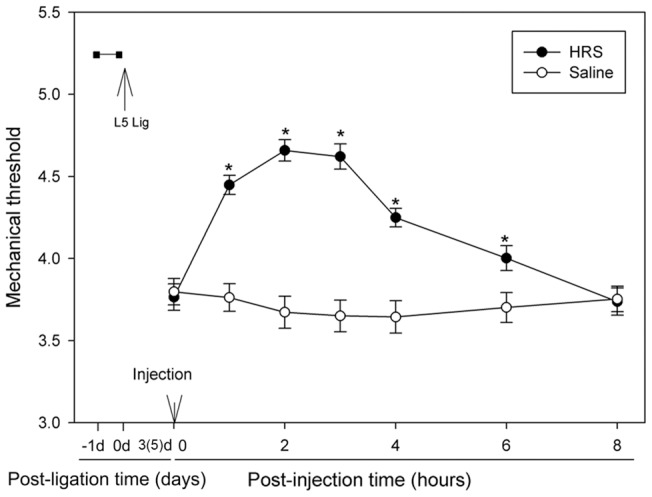
Effects of intrathecal injection of HRS on mechanical thresholds in rat model of neuropathic pain. On the third post-operative (PO) day, the rats were divided into two groups of five, each group receiving either HRS or NS, and behavior tests were done for 8 hrs followed by 48 hrs of rest, and the testing process was repeated. So every rat received all the two treatments followed by behavior testing after each. Thus each point represents the results from ten rats. Note the great increase of mechanical thresholds after HRS injections. Eight hrs after the injection, the thresholds declined to the level of the NS group. Data are expressed as mean±SEM. n = 10. Asterisks indicate significant (p<0.05) differences from the NS control group by a two-way repeated ANOVA.

Mechanical thresholds of neuropathic rats were evaluated after intrathecal injection of single-dose HRS or NS using a randomized cross-over design [7]. L5 SNL was performed on 10 rats, a procedure that resulted in marked reduction in mechanical thresholds. On the third post-operative (PO) day, the rats were divided into two groups of five, each group receiving either HRS or saline. Mechanical thresholds were determined for all animals at a set of time points (1 hr, 2 hrs, 3 hrs, 4 hrs, 6 hrs, 8 hrs after the injection. The analgesic effect of HRS was short-acting, these time points were chosen to show the pharmacodynamic characters). All animals were then rested for 48 hrs. On the fifth PO day, each group received another injection of HRS or NS, which they did not receive before, followed by the same paradigm of behavioral testing. Figure 1 show the summary of all the testing results and each point represents tests on 10 rats. Note that mechanical thresholds were markedly reduced after nerve ligation, and administration of HRS raised these thresholds for about 8 hrs before the thresholds declined to the level of the NS group.

### The long-term effect of HRS treatment on mechanical allodynia and thermal hyperalgesia

**Figure 2:**
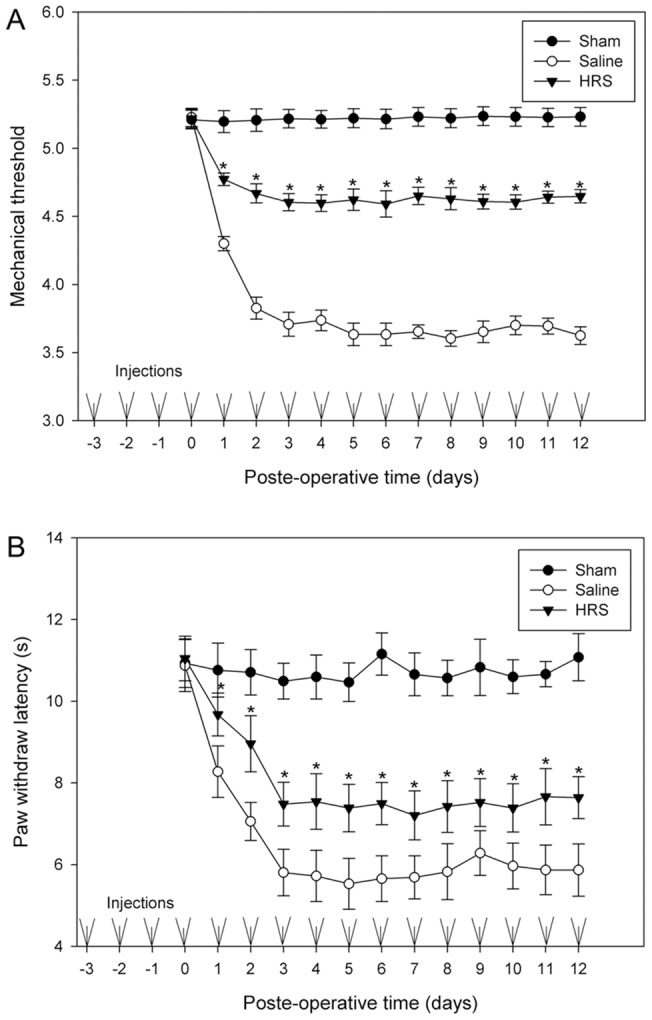
Effects of repeated injection of HRS on mechanical thresholds and paw withdraw latency. Staring 3-operation rats received no injection. Mechanical thresholds (A) and PWLs (B) of all rats were determined every 24 hrs. Mechanical thresholds and PWLs of HRS or NS treated rats remained the same levels as the sham rats at the time point of 0 PO day. Note the great increase in mechanical thresholds and PWLs of rats received HRS, and that there is no development of tolerance. Data are expressed as mean±SEM. n = 10 for A and B. Asterisks indicate values significantly (p<0.05) different from controls using a two-way repeated ANOVA followed by the Duncan post hoc test.

To examine long-term effects of HRS, 20 rats were selected and randomly divided into two groups: HRS group and NS group. These 20 rats received intrathecal injection of HRS or saline respectively at 24 hrs intervals for 15 days, on the third day after the first dose, the L5 spinal nerve was ligated. Another 10 rats were selected and underwent a sham operation but received no injection. From the L5 SNL day to 12 days after L5 SNL, mechanical thresholds and paw withdraw latencies (PWLs) were determined for all animals at the time points 23–24 hrs after each injection, the first examinations of mechanical threshold and PWL for each rat were done before L5 SNL. Figure 2 shows the results of behavior testing. Note that mechanical thresholds and PWLs of HRS or NS treated rats remained the same levels as the sham rats at the time point of 0 PO day. It was found that mechanical thresholds and PWLs were reduced dramatically after L5 SNL. Mechanical thresholds of neuropathic rats received HRS were increased to a constant level, which was significantly higher than rats received NS (Figure 2 A). Repeated administration of HRS markedly raised PWL of the neuropathic rats, and no significant fluctuations were observed when PWLs reached a latency of 7.5 seconds (on the third PO day). NS injection did not affect the marked decline of mechanical thresholds or PWLs (Figure 2 B). We concluded that no tolerance develops when animals receive repeated treatment of HRS, and injections of HRS do not affect the basal mechanical threshold and PWL in rat without nerve ligation.

### The effect of preemptive HRS treatment on mechanical allodynia and thermal hyperalgesia

**Figure 3:**
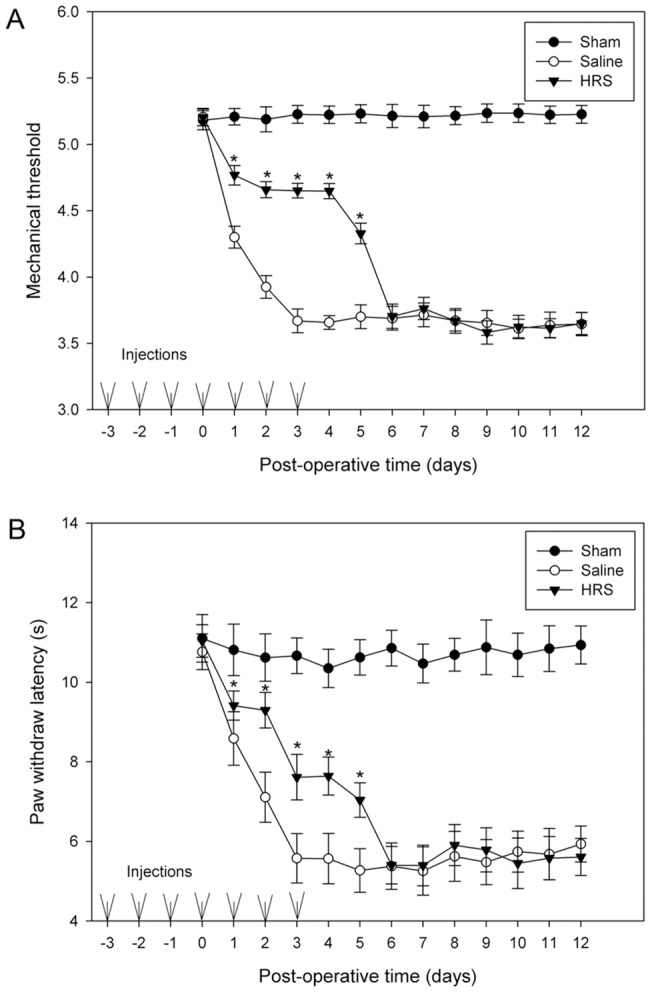
Effects of HRS pretreatment on the development of neuropathic pain behavior. All rats received repeated injection of HRS or NS at 24(A) and PWLs (B) were significantly elevated in HRS group as compared to controls, and this effect lasted for 3 days after the administration ceased. Data are expressed as mean±SEM. n = 10 for A and B. Asterisks indicate values significantly (p<0.05) different from controls using a two-way repeated ANOVA followed by the Duncan post hoc test.

To examine preemptive analgesic effects, starting 3 days prior to the neuropathic lesion, HRS was administered intrathecally to 10 rats at 24 hrs intervals for 6 days. Additional 10 rats received the same paradigm of saline injection, serving as a control group. On the third day after the first dose, the L5 spinal nerve was ligated. 10 rats that underwent a sham procedure received no injection. From the L5 SNL day to 12 days after L5 SNL, mechanical thresholds and PWLs were determined for all animals at the time points 23–24 hrs after each injection. Figure 3 is a summary of the behavior testing results. Evident is the slow decline in mechanical thresholds after the lesion in rats of the HRS group, and that the thresholds remain at a higher level than saline group for several days after cessation of HRS. Mechanical thresholds in neuropathic rats treated with saline declined sharply, and then became stable when it reached the level of 3.67 (in logarithmic units). Mechanical thresholds in rats of the sham group did not change significantly after the operation (Figure 3 A). The mean PWL was 5.21±0.67 seconds (mean±SD) in rats of sham group and did not change significantly during the test. Ligation of the spinal nerve caused a significant decrease in PWLs in rats receiving daily NS treatments. PWLs declined much slowly after the lesion in rats receiving daily HRS treatments, and PWLs remain at a higher level than NS group for several days after cessation of HRS (Figure 3 B), indicating that HRS treatment provided a relatively long-lasting preemptive effect.

### Astrocytic responses to L5 SNL in NS treated neuropathic rats and HRS treated neuropathic rats

**Figure 4:**
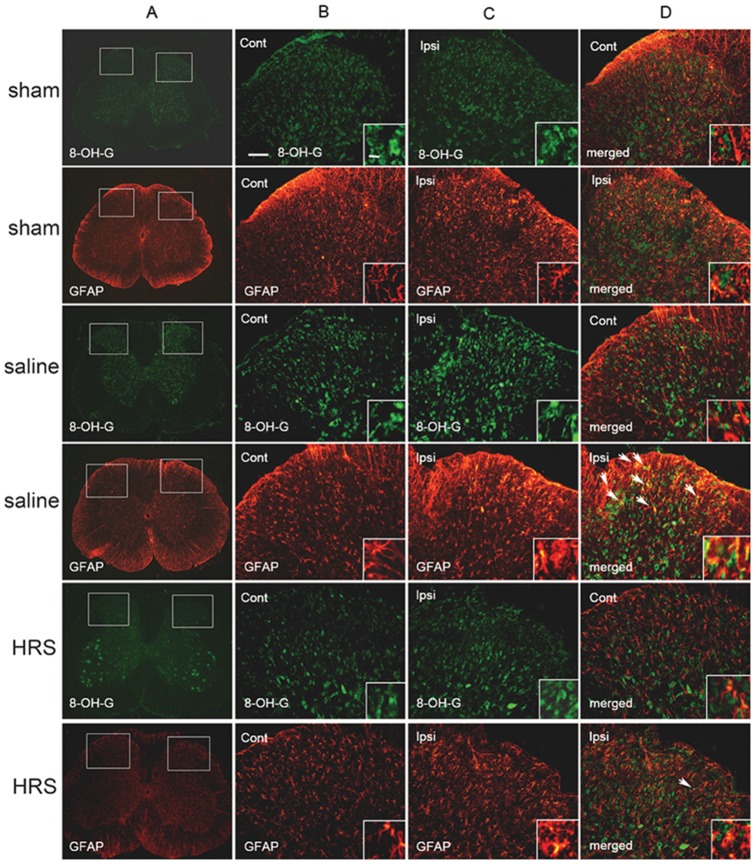
Immunostaining for 8-OH-G and GFAP in sham rats, NS-treated neuropathic rats and HRS-treated neuropathic rats. Pictures in columns B and C are magnification of the insets shown in column A. Enlargements in the insets in columns B, C and D demonstrate the changes in cell shape and expression of 8-OH-G (green) and GFAP (red). Double-staining demonstrate colocalization (arrows) of 8-OH-G and GFAP. Note that an increased number of cells showing immunoreactivity for 8-OH-G and GFAP in neuropathic rats treated with NS as compared to the sham rats. And number of cells showed colocalization of 8-OH-G and GFAP in NS treated neuropathic rats was significantly increased as compared to the sham rats. HRS greatly reversed L5 SNL induced production of 8-OH-G, activation of astrocytes, and increase of 8-OH-G-ir astrocytes. Scale bars, 100 µm and 25 µm in the inset.

**Figure 5:**
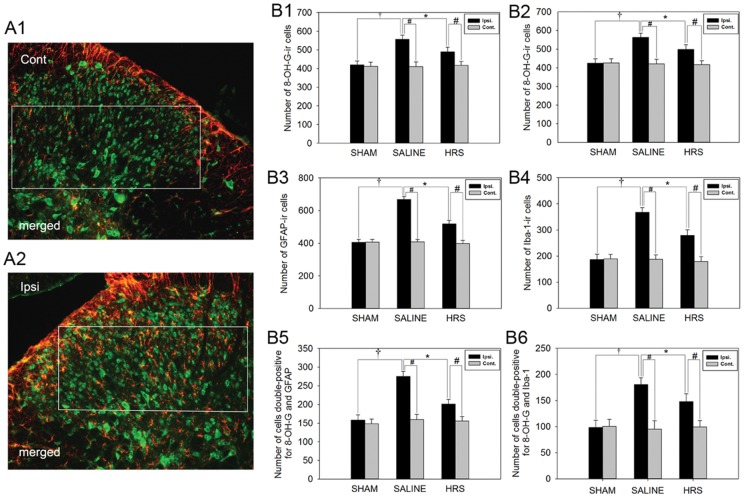
Numbers of cells expressing 8-OH-G, GFAP, Iba-1 and numbers of double-stained cells in all rats. (A1, A2) Merged images of photomicrographs immunostaining for 8-OH-G (green) and GFAP (red) in L5 sections of neuropathic rats treated with NS were taken as example, a rectangular area was manually outlined in the dorsal horn using the rectangular marquee tool, and the size of every selected area is 50 cm×22 cm (2500 µm×1100 µm in the spinal sections). At 20-fold magnification, counts were obtained of the numbers of cells in the areas outlined in the rectangular that were positive for 8-OH-G, GFAP and Iba-1, double staining cells in the areas were also counted. (B1, B2) Number of cells showing immunoreactivity for 8-OH-G in both sides of spinal dorsal horn. (B3, B4) Number of cells showing immunoreactivity for GFAP and Iba-1 in both sides of spinal dorsal horn. (B5, B6) Number of 8-OH-G-ir astrocytes and 8-OH-G-ir microglia in both sides of spinal dorsal horn. Data are expressed as mean±SEM. n = 10 for B1-6. Compared with the sham rats, L5 SNL induced significant production of 8-OH-G, activation of glial cells and increase of 8-OH-G-ir glial cells, †p<0.01, by one-way ANOVA. HRS blocked the production of 8-OH-G, glial activation and increase of 8-OH-G-ir glial cells, *p<0.01, by one-way ANOVA. Compared to contralateral side, L5 SNL induced significant production of 8-OH-G, activation of glial cells and increase of 8-OH-G-ir glial cells in ipsilateral side. #p<0.01, by one-way ANOVA.

To examine whether HRS could alleviated oxidative stress in the spinal dorsal horn, and reverse the activation of spinal astrocytes, 8-OH-G and glial fibrillary acidic protein (GFAP) immunoreactivities were assessed in the lumbar spinal cord of sham rats, L5 SNL rats treated with HRS and L5 SNL rats treated with NS. To identify whether 8-OH-G was produced in astrocytes, we performed double immunofluorescence staining of 8-OH-G with GFAP. Rats were sacrificed 1 hr after HRS or NS treatment, on the 12th day after L5 SNL or sham operation. In the L4-L5 lumbar dorsal horns of rats in the sham group, low-level 8-OH-G immunostaining was observed. In the neuropathic rats treated with NS, levels of 8-OH-G on the ipsilateral side of spinal dorsal horns were markedly higher, and HRS treated neuropathic rats showed lower production of 8-OH-G in the lumbar dorsal horn compared with NS treated neuropathic rats, as shown in Figure 4 and Figure 5 B1, B2.

In the sham group, the immunostaining of GFAP was low, and the stained astrocytes had extensive processes and were well spaced showing no obvious signs of astrocytic activation. In the neuropathic rats treated with NS, spinal astrocytes on the ipsilateral side were intensely stained and appeared to have an altered shape with long protrusions showing an activated state. In the neuropathic rat treated with HRS, the immunostaining of GFAP was lower, and showed less astrocytic activation, as compared with the neuropathic rats treated with NS (Figure 4 and Figure 5 B3).

Double immunofluorescence staining revealed that spinal astrocytes of sham rats produced a small amount of 8-OH-G. In the spinal cord of neuropathic rats treated with NS, an increased number of astrocytes showed colocalization of 8-OH-G and GFAP on the ipsilateral side. HRS administration lowered the number of astrocytes showing colocalization of 8-OH-G and GFAP (Figure 4 and Figure 5 B5).

### Microglial responses to L5 SNL in NS treated neuropathic rats and HRS treated neuropathic rats

**Figure 6:**
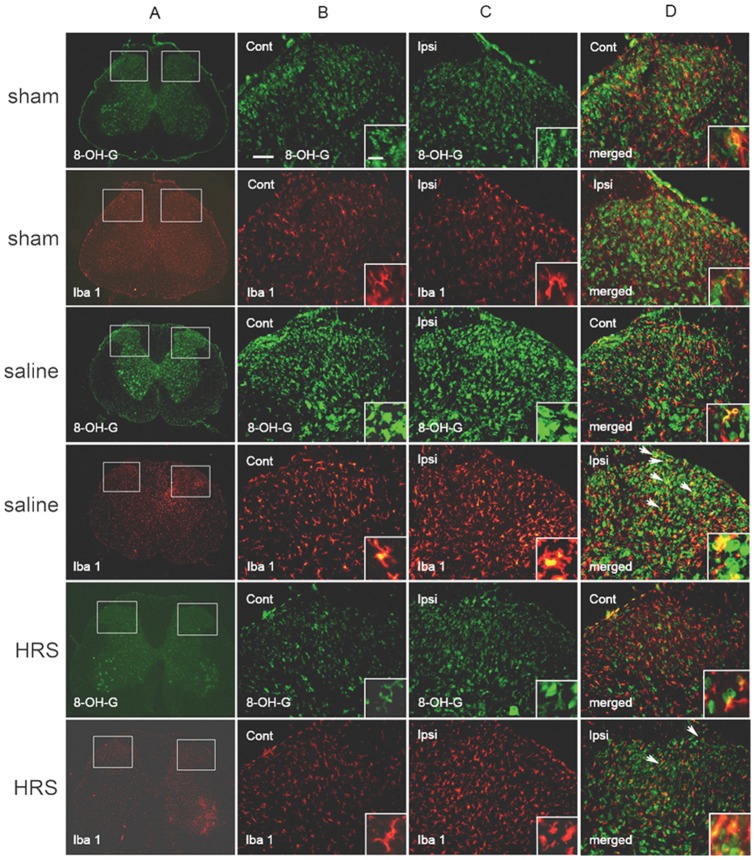
Immunostaining for 8-OH-G and Iba1 in sham rats, NS-treated neuropathic rats and HRS-treated neuropathic rats. Pictures in columns B and C are magnification of the insets shown in column A. Enlargements in the insets in columns B, C and D demonstrate the changes in cell shape and expression of 8-OH-G (green) and Iba-1 (red). Double-staining demonstrate colocalization (arrows) of 8-OH-G and Iba1. Note that an increased number of cells showing immunoreactivity for 8-OH-G and Iba-1 in neuropathic rats treated with NS as compared to the sham rats. And number of cells showed colocalization of 8-OH-G and Iba-1 in NS treated neuropathic rats was significantly increased as compared to the sham rats. HRS greatly reversed L5 SNL induced production of 8-OH-G, activation of microglia, and increase of 8-OH-G-ir microglia. Scale bars, 100 µm and 25 µm in the inset.

The ionized calcium-binding adapter molecule 1 (Iba-1) level in the spinal dorsal horn of sham rats, L5 SNL rats treated with HRS and L5 SNL rats treated with NS were examined to determine whether HRS could alleviate the activation of spinal microglia. Double immunofluorescence staining of 8-OH-G with Iba-1 was also performed to identify whether 8-OH-G was produced in microglia. Rats were sacrificed 1 hr after HRS or NS treatment, on the 12th day after L5 SNL or sham operation. In the sham rats, the Iba-1 immunostaining was low, and microglia stained with Iba-1 showed features of an inactivated state. They had extensive and thinly branched processes and were well spaced. In neuropathic rats treated with NS, microglia on the ipsilateral side were heavily stained and exhibited short, thick processes and enlarged cell bodies, showing characteristics of an activated state. These phenotypic features of resting microglia and activated microglia are consistent with previous studies [41], [42]. In neuropathic rats treated with HRS, microglia on the ipsilateral side were activated, but showed fewer features of an activated state than neuropathic rats treated with NS (Figure 6 and Figure 5 B4).

Double immunofluorescence staining revealed that spinal microglia in sham rats produced a small amount of 8-OH-G (Figure 6 and Figure 5 B6). In neuropathic rats treated with NS, the number of microglia on the ipsilateral side of the dorsal horn that showed colocalization of 8-OH-G and Iba-1 was significant larger compared with sham rats. And in neuropathic rats treated with HRS, the number of microglia showing colocalization of 8-OH-G and Iba-1 on the ipsilateral side was larger than that of the sham group, but less than that of the animal treated with saline (Figure 6 and Figure 5 B6).

### Rats received catheterization demonstrate normal basal expression of 8-OH-G and normal activation of glial cells in spinal dorsal horn

**Figure 7:**
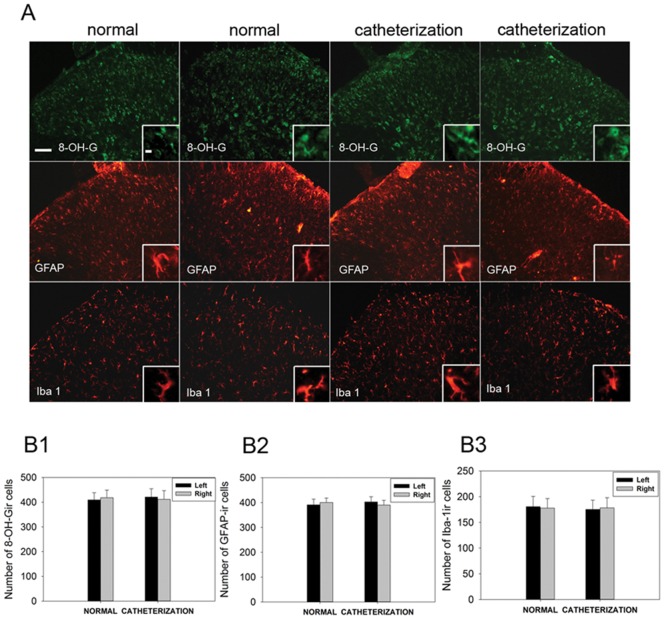
Expression of 8-OH-G, GFAP and Iba-1 in catheterized rats. (A) Photomicrographs of immunostaining for 8-OH-G (green), GFAP (red) and Iba1 (red) in normal rats and catheterized rats. Enlargements in the insets demonstrate the changes in cell shape and expression of 8-OH-G, GFAP and Iba-1. There is no significant difference in number of cells showing immunoreactivity for 8-OH-G, GFAP and Iba-1 between catheterized rats and normal rats. Scale bars, 100 µm and 25 µm in the inset. (B1, B2 and B3) Number of cells showing immunoreactivity for 8-OH-G, GFAP and Iba-1 in both sides of spinal dorsal horn. Data are expressed as mean±SEM. n = 10 for B1-3. Compared to the normal rats, catheterization did not induced significant production of 8-OH-G, activation of glial cells.

To exclude the fact that possible over-expression of 8-OH-G and activation of glial cells are caused by catheterization, 8-OH-G, GFAP, and Iba-1 immunoreactivities were assessed in the lumbar spinal cord of normal rats and rats received catheterization 15 days before. In the L4-L5 lumbar dorsal horns, low-level 8-OH-G, GFAP and Iba-1 immunostaining were observed in both normal rats and catheterized rats (Figure 7A). Spinal glial cells had cell bodies that were small and elongated with long protrusions showing no obvious signs of activation. Numbers of cells showing immunoreactivity for 8-OH-G, GFAP and Iba-1 were comparable in normal rats and catheterized rats (Figure 7 B1, B2 and B3).

### HRS reverses the elevated tyrosine-nitrated Mn-containing superoxide dismutase (MnSOD) expression in spinal cord of L5 SNL rats

**Figure 8:**
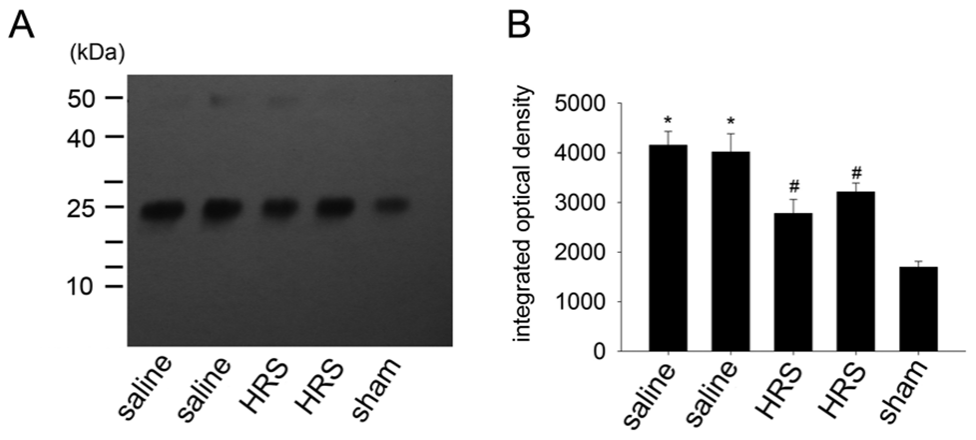
HRS blocks nitrition of MnSOD in spinal cord of neuropathic rats. (A) When compared to the sham group (lane 5), L5 SNL induced significant nitration of MnSOD in neuropathic rats treated with NS (lane 1,2). These events were blocked by HRS treatment (lane 3,4). Approximate sizes (kDa) were estimated using prestained molecular mass markers to the left. (B) The integrated optical density (IOD) of lanes resulted from immunoprecipitation was obtained using Image-pro plus analysis. Data are expressed as mean±SEM (n = 5). Compared to the sham rats, L5 SNL induced significant production of tyrosine-nitrated MnSOD, *p<0.01, by one-way ANOVA. HRS blocked the production of tyrosine-nitrated MnSOD, #p<0.01, by one-way ANOVA.

Rats were sacrificed 1 hr after HRS or NS treatment, on the 12th day after L5 SNL or sham operation. Nitration of tyrosine residues in MnSOD of the lumbar dorsal horn was detected by Western blot analysis of SDS/PAGE-fractionated tyrosine-nitrated proteins, which had been immunoprecipitated from total spinal extracts using the monoclonal anti-nitrotyrosine. Immunodetection with monoclonal anti-MnSOD antibody demonstrated low but detectable levels of tyrosine-nitrated MnSOD in sham rats. Neuropathic rats treated with NS exhibited an increased level of immunoreactive tyrosine-nitrated MnSOD, while neuropathic rats treated with HRS exhibited a lower level of tyrosine-nitrated MnSOD as compared with neuropathic rats treated with NS, though it was higher than that in sham rats (Figure 8).

### HRS reduces the expression of proinflammatory cytokines in the lumbar spinal cord

**Figure 9:**
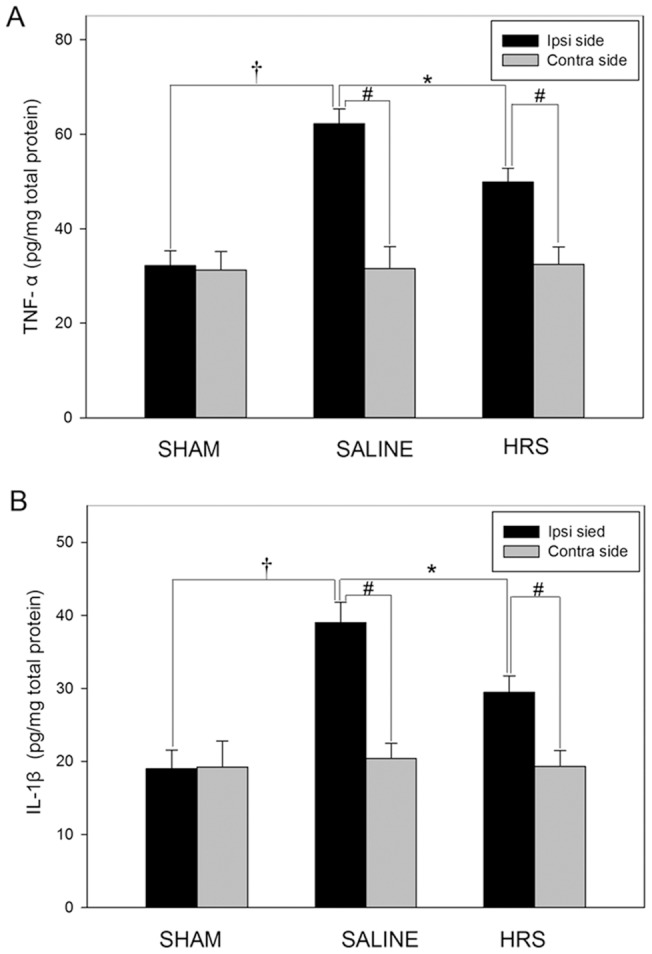
Protein content of TNF-α (A) and IL-1β (B) in both sides of spinal dorsal horn. Data are expressed as mean±SEM. n = 10 for A and B. Compared to the sham rats, L5 SNL induced significant production of TNF-α and IL-1β, †p<0.01, by one-way ANOVA. HRS blocked the production of TNF-α and IL-1β, *p<0.01, by one-way ANOVA. Compared to contralateral side, L5 SNL induced significant production of TNF-α and IL-1β in ipsilateral side. #p<0.01, by one-way ANOVA.

To investigate whether the analgesic effect of HRS was associated with the decrease in proinflammatory cytokines production, protein levels of TNF-α and IL-1β on both sides of the spinal cord were assessed using enzyme-linked immunosorbent assay (ELISA). 12 days after nerve ligation, the spinal expressions of both TNF-α and IL-1β on the ipsilateral side of rats treated with NS were up-regulated in comparison with the sham-operated rats (TNF-α: p<0.01; IL-1β: p<0.01). While HRS reversed the over-expressions (TNF-α: p<0.01; IL-1β: p<0.01)(Figure 9 A and B). The spinal expressions of TNF-α and IL-1β on the contralateral side of rats treated with saline did not differ from those in the sham group. In comparison with the contralateral side, expressions of TNF-α and IL-1β on the ipsilateral side of the spinal cord of rats treated with NS were found also up-regulated, while HRS reversed the over-expressions (TNF-α: p<0.01; IL-1β: p<0.01)(Figure 9 A and B).

## Discussion

This study showed that intrathecal HRS significantly reduced the behavior signs of allodynia and hyperalgesia in neuropathic rats. Administration of single-dose HRS attenuated mechanical allodynia for about 8 hrs, repeated treatment of HRS ameliorated mechanical allodynia and thermal hyperalgesia without developing tolerance. Preemptive HRS treatment had a long-last effect on mechanical allodynia and thermal hyperalgesia.

In the L4-5 spinal dorsal horn, L5 SNL induced increased production of 8-OH-G, and significantly activated astrocytes and microglia. Intrathecal HRS reduced L5 SNL-induced over-production of 8-OH-G and attenuated activation of glial cells. 8-OH-G production was found in astrocytes and microglia, as indicated by immunostaining. Glial cells activated concurrently when 8-OH-G in glial cells was over-produced, on the contrary, glial activation was attenuated when 8-OH-G was eliminated by HRS. The hydroxyl radical is able to add to double bonds of DNA bases at a second-order, abstracts an H-atom from the methy group and leads to formation of 8-OH-G. Our observation of over-produced 8-OH-G in activated glial cells and reduced 8-OH-G production in less activated glial cells suggests a mechanism of enlargement of pain signals from primary afferent neurons by hydroxyl activated glial cells which play a key role in modulation of pain signals.

Peroxynitrite can nitrate the 3-position of tyrosine residues on MnSOD, indicating that tyrosine-nitrated MnSOD level is directly related to peroxynitrite. We have shown in this study that L5 SNL increased the production of tyrosine-nitrated MnSOD in the lumbar dorsal horn, and intrathecal HRS significantly reduced the over-production of tyrosine-nitrated MnSOD and attenuated allodynia and hyperalgesia. Therefore, we conclude that peroxynitrite contributes to the enhanced processing of nociceptive messages in spinal dorsal horn. Salvemini and coworkers have demonstrated that peroxynitrite plays a key role in nitroxidative regulation of the glutamatergic pathway during nociceptive processing, modulation of glutamate transmission by tyrosine residues nitration, and enhanced neuroimmune activation [43]–[45]. Our study showed that intrathecal HRS significantly attenuated L5 SNL-induced activation of spinal glial cells, suggesting that spinal peroxynitrite might be involve in the activation of glial cells, and leads to neuropathic pain.

It has been demonstrated that both astrocytes and microglia are capable of releasing proinflammatory factors [46]–[48]. In the present study, protein levels of TNF-α and IL-1β on both sides of the spinal cord were assessed. 12 days after L5 SNL, expression of TNF-α and IL-1β on the ipsilateral side were significantly up-regulated in comparison with the sham-operated rats, while HRS reversed their over-expression, indicating that changes in levels of proinflammtory factors expression are correlated with the changes in level of hydroxyl and peroxynitrite.

Chronic pain is a state where abnormalities are maintained in both central and peripheral nervous systems [49]. In the spinal cord, there are multiple potential sites where ROS and RNS can exert effects contributing to neuropathic pain, include three mechanisms implicated in central sensitization: alteration in glutamateric neurotransmission, loss of tonic inhibitory controls and glial-neuronal interactions [50], here we focus on glial-neuronal interactions. Under normal conditions, microglia functions as resident macrophages in the CNS. Within hours of peripheral nerve injury, activated microglia accumulates in the superficial dorsal horn. The activated microglial releases proinflammatory cytokines (such as TNF-α, IL-1β and 6), leading to neuronal central sensitization. In the present study, we showed that L5 SNL induced significant activation of microglia mediated by hydroxyl and peroxynitrite and up-regulated the expression of TNF-α and IL-1β on the ipsilateral side. In addition to proinflammatory cytokines, Coull and coworkers demonstrated that activated microglia induced by P2X4 receptors released brain-derived neurotrophic factor (BDNF) [26]. The contribution of astrocytes to central sensitization remains unclear. Many studies showed that activated astrocytes played an important role in central sensitization [51]–[53], and unlike microglia, astrocyte activation is generally delayed and persists longer, up to several months [50]. Our study showed that L5 SNL induced significant activation of spinal astrocytes mediated by hydroxyl and peroxynitrite.

The selective vulnerability of neural systems to ROS is a common characteristic of age-related degenerative diseases and some painful nerve conditions (including neuropathic pain) [8], [54]–[59]. Knowing pain perception is amplified when noxious stimuli continue and oxidative stress in spinal cells is enhanced in neuropathic conditions [7], [60], we postulate that on-going afferent discharges in persistent pain states elevates ROS and RNS production in the spinal cord, and lead to abnormal activation of spinal cells. In the present study, 12 days after L5 SNL, an increased production of ROS and RNS was detected in the spinal dorsal horn, and significant activation of astrocytes and microglia was observed. In the spinal cord of rats treated with HRS, levels of ROS and RNS were lower, and fewer activated glial cells were detected, and the behavior signs of allodynia and hyperalgesia were reduced. Allan and coworkers stated that under persistent pain state, the processes of central sensitization medicated by activated of N-Methyl-D-aspartate (NMDA) receptor are comparable to that implicated in the plastic changes associated with hippocampal long-term potentiation (LTP), a cellular correlate for learning and memory. Jin Mo Chung have demonstrated that ROS were involved in LTP in spinal dorsal horn [61]. In our opinion, ROS and RNS may be key molecules involved in the spinal LTP in neuropathic rats.

Although antioxidants are known to be potentially effective for treatment of neuropathic pain, concerns about their adverse effects, half-life, and tolerance remain. PBN is a potent ROS scavenger, Kim and coworkers showed that PBN ameliorated the behavioral signs of mechanical allodynia in rat model [7]. However, although this compound proved to be a neuroprotective agent, it induced hypothermia and toxicity [62]. Hydrogen gas cannot be produced by the human body since mammalian cells lack the hydrogenase activity [63]. However, it is continuously produced by colonic bacteria in the body and normally circulates in the blood [64]. Studies showed that inhalation of hydrogen gas did not influence physiological parameters such as body temperature, blood pressure, pH, and PO2 in the blood [65]. The half-life of HRS injected intrathecally has not been established yet the analgesic effect persisted for about 8 hrs. Fortunately, no tolerance developed when animals were treated with HRS repeatedly. So, HRS has the following advantages: easy to use, no side effects and no tolerance develops when given continuously, indicating that therapeutic use of HRS may be promising. HRS infusion and H_2_ inhalation are most commonly used in clinic hydrogen treatment. Hydrogen administration may not be therapeutic unless enough concentration stays in the spinal cord, so the effective doses needed should be determined before clinical use.

## Conclusions

Intrathecal injection of HRS produced analgesic effect in the rat model of neuropathic pain. Preemptive treatments with HRS delayed the development of pain behavior, and no tolerance developed when HRS was given repeatedly. The side effect of HRS seems tenuous as HRS selectively reduces hydroxyl and peroxynitrite. HRS reduced the intracellular levels of hydroxyl and peroxynitrite in the spinal cord, blocked the activation of spinal glial cells, and reduced the expression of proinflammatory factors in the spinal cord. We conclude that abnormal elevation of ROS and RNS plays a key role in central sensitization under neuropathic conditions. HRS has a potentially promising prospect for clinical treatment of neuropathic pain.
