# Correction: Intrathecal Infusion of Hydrogen-Rich Normal Saline Attenuates Neuropathic Pain via Inhibition of Activation of Spinal Astrocytes and Microglia in Rats

**DOI:** 10.1371/journal.pone.0109482

**Published:** 2014-09-23

**Authors:** The PLOS ONE Staff

There is an error in affiliation 1 for authors Yanhu Ge, Feixiang Wu, Liqun Yang, Zhijie Lu, Yuming Sun, and Wei-Feng Yu. Affiliation 1 should be: Department of Anesthesiology, Eastern Hepatobiliary Surgery Hopsital, Second Military Medical University, Shanghai, China.
